# Saunders’s Medical Hand-Atlases

**Published:** 1905-03

**Authors:** 


Saunders's Medical Hand-Atlases. Atlas and Epitome of General Pathologic Histology. By Dr. H. Durck, Munich. Edited, with additions, by Ludvig Hektoen, M.D., Professor of Pathology, Rush Medical College, Chicago. With 172 colored figures on 77 lithographic plates, 36 text-cuts, many in colors, and 371 pages of text. Philadelphia, New York, London: W. B. Saunders & Company. 1904. (Cloth, $5.00 net.)

Judged from purely artistic standpoints this latest issue of Saunders's series of atlases is one of the most exquisitely finished publications ever turned out. In the reviews of previous volumes of these handbooks especial mention has been made of the uniform excellence of the illustrative features, upon which much time and thought have been spent. Every detail of the subject under consideration has been brought forth and, however brilliant and learned the text, the illustrations have been the chief value of each book. Without them the volumes would be merely well-written essays upon a special subject. So, too, would be the atlas of general pathologic histology,—a most learned presentation, clear, concise, understandable; but with the illustrations it becomes even more; it is rather a complete laboratory course with every pathologic condition, shown just as clearly, just as "life-like," as if one were examining the freshly-prepared specimen under a microscope.

In this, as in previous volumes, there is painstaking avoidance of exhaustive critical consideration. The many fads and fancies which find place in all branches are carefully ignored and only that is presented which is helpful and instructive, and in just the lucid manner which marked the previously issued volumes on special pathologic histology. The illustrations, numbering 176 in colors on 80 lithographic plates, and 36 figures in black and white, are from original specimens and everything has been sacrificed,—time and money,—to secure absolute fidelity in reproduction of the original water colors, and the result is marvelously beautiful, some of the plates combining 26 colors for the perfect reproduction and the demonstration of minute microscopic detail.

Dr. Hektoen has done an excellent work in editing the volume and has added much valuable material. There is no work on general pathologic histology published in the English language which can be compared with this or even approach it in any of its phases.

